# ReDirection: an R-package to compute the probable dissociation constant for every reaction of a user-defined biochemical network

**DOI:** 10.3389/fmolb.2023.1206502

**Published:** 2023-10-24

**Authors:** Siddhartha Kundu

**Affiliations:** Department of Biochemistry, All India Institute of Medical Sciences, New Delhi, India

**Keywords:** biochemical network, null space-generated subspaces of combinatorial sums of non-trivial and nonredundant vectors, probable disassociation constant and reaction outcome, “R”-package, reaction-specific sequence and outcome vectors, stoichiometry number matrix

## Abstract

Biochemical networks integrate enzyme-mediated substrate conversions with non-enzymatic complex formation and disassembly to accomplish complex biochemical and physiological functions. The choice of parameters and constraints used in most of these studies is numerically motivated and network-specific. Although sound in theory, the outcomes that result depart significantly from the intracellular milieu and are less likely to retain relevance in a clinical setting. There is a need for a computational tool which is biochemically relevant, mathematically rigorous, and unbiased, and can ascribe functionality to and generate potentially testable hypotheses for a user-defined biochemical network. Here, we present “ReDirection,” an R-package which computes the probable dissociation constant for every reaction of a biochemical network directly from a null space-generated subspace of the stoichiometry number matrix of the modeled network. “ReDirection” delineates this subspace by excluding all trivial and redundant or duplicate occurrences of non-trivial vectors, combinatorially summing the vectors that remain and verifying that the upper or lower bounds of the sequence of terms formed by each row of this subspace belong to the open real-valued intervals $\left(-\infty ,-1\right)$ or $\left(1,\infty \right)$ or whether the number of terms that are differently signed are almost equal. “ReDirection” iterates these steps until these bounds are consistent and unambiguous for all reactions of the modeled biochemical network. Thereafter, “ReDirection” filters the terms from each row of this subspace, bins them to outcome-specific subsets, sums and maps this to an outcome-specific reaction vector, and computes the p1-norm, which is the probable dissociation constant for a reaction. “ReDirection” works on first principles, does not discriminate between enzymatic and non-enzymatic reactions, offers a biochemically relevant and mathematically rigorous environment to explore user-defined biochemical networks under baseline and perturbed conditions, and can be used to address empirically intractable biochemical problems. The utility and relevance of “ReDirection” are highlighted by numerical studies on stoichiometric number models of biochemical networks of galactose metabolism and heme and cholesterol biosynthesis. “ReDirection” is freely available and accessible from the comprehensive R archive network (CRAN) with the URL (https://cran.r-project.org/package=ReDirection).

## 1 Introduction

An undirected biochemical network is converted into a pathway by a combination of physicochemical (temperature, pH, and compartmentalization) and biochemical (small-molecule effectors, shared intermediates, and feedback) factors. Despite the availability and accessibility of advanced data analytical tools, true mechanistic insights into the manner in which a biochemical network accomplishes a complex function are unclear (Ferrara et al., 2008; Keller and Attie, 2010; Biane and Delaplace, 2019; Seyhan and Carini, 2019; Koutrouli et al., 2020). An essential first step in the analysis of a biochemical network is the construction of a suitable model. This is usually data-driven and coarse-grained, where nodes can represent proteins, genes, or cells, and edges indicate lines of supporting evidence (empirical, “omics” datasets, co-expression data, text mining, and knowledge-based databases) (Reinker et al., 2006; Ferrara et al., 2008; Lecca et al., 2009; Keller and Attie, 2010; Haraldsdottir et al., 2012; Shindo et al., 2018; Biane and Delaplace, 2019; Seyhan and Carini, 2019; Koutrouli et al., 2020; Wittenstein et al., 2022). Analyzing such a network results in several network-specific characteristics such as the clustering coefficient and path distance (Reinker et al., 2006; Lecca et al., 2009; Haraldsdottir et al., 2012; Shindo et al., 2018; Wittenstein et al., 2022). This initial characterization can be complemented by a library of equally plausible outcomes, all of which are made to approximate the original architecture (Lecca et al., 2009; Riva et al., 2022). Inverse modeling, for a dataset, generates several possible candidate causal network models, allows hypothesis testing, and may potentially be more informative (Reinker et al., 2006; Lecca et al., 2009; Haraldsdottir et al., 2012; Rottman and Hastie, 2014; Shindo et al., 2018; Riva et al., 2022; Wittenstein et al., 2022).

Causal networks (CNs) are probability-based and can model alternate scenarios for every node of a small network whilst concomitantly ascribing specific states to each node (Rottman and Hastie, 2014). Although CNs have had considerable success in investigating real-world problems, inferring biochemical function from a network of genes/proteins/metabolites remains challenging (Rottman and Hastie, 2014). For example, a causal network is usually modeled as an “acyclic” graph, which is in complete contrast to the plethora of feedback (positive and negative) mechanisms and reverse reactions that exemplify biochemical systems (Rottman and Hastie, 2014). CNs are also inferential, modeled as a homogenous Poisson’s process (discrete event, discrete domain) and inherently Markovian (Lecca et al., 2009; Rottman and Hastie, 2014). Biochemical function, on the other hand, is dependent on thresholds (signal transduction and pattern receptors), characterized by minor perturbations and is memory-driven, all of which are better modeled as continuous events or variables in discrete time. CNs, to be truly informative, also require a significant amount of initial data, which is a major limitation in modeling biochemical networks. These arguments notwithstanding, CNs have contributed to well-defined observables in the presence of ample empirical data, such as phenotype mapping, along with dose- and stimulus-driven response of genes (Goto et al., 2019; Gopalan et al., 2021; Lu et al., 2021; Salvador et al., 2021; Saptarshi et al., 2021). CNs of genes and proteins result in lists which can be utilized for large-scale data mining (parameter selection and candidate genes) and/or analytics, as in precision medicine and biomarker profiling (Biane and Delaplace, 2019; Goto et al., 2019; Seyhan and Carini, 2019; Gopalan et al., 2021; Lu et al., 2021; Salvador et al., 2021; Saptarshi et al., 2021).

Unlike data-driven modeling, optimization- and enumeration-based strategies can be used to investigate and characterize a biochemical network from first principles and at the near-steady state (Segre et al., 2002; Shlomi et al., 2005; Wagner and Urbanczik, 2005; Urbanczik, 2007; Orth et al., 2010; Muller and Regensburger, 2016; Klamt et al., 2017; Klamt et al., 2018; Lee et al., 2020). Algorithms which assess the flux of a reactant (flux balance analysis, flux variability analysis, regulatory on–off minimization, and minimization of metabolic adjustment) will maximize or minimize the biomass of a metabolite of interest and can be used to investigate the effects of deletions and other perturbations on the flux of metabolites through a large network (Segre et al., 2002; Shlomi et al., 2005; Orth et al., 2010; Klamt et al., 2018; Lee et al., 2020). The numerical enumeration of elementary flux modes and vectors, along with extreme pathway analysis, can be used to derive meaningful information about “metabolic” hubs and smaller subsets of cooperating reactions from biochemical networks (Wagner and Urbanczik, 2005; Urbanczik, 2007; Muller and Regensburger, 2016; Klamt et al., 2017). A mathematical model of a biochemical network can also be made to integrate real-time data such as from “omics”-based studies, spectroscopic analysis, and pulse-chase experiments, which allows an investigator to refine and optimize the model (Antoniewicz, 2015; Heuillet et al., 2018; Wang et al., 2020). This approach of combining experimental data with theoretical studies is referred to as metabolic flux analysis (MFA) and is utilized in biotechnological applications to regulate the biomass of a preferred reactant/product (Antoniewicz, 2015; Heuillet et al., 2018; Wang et al., 2020).

The aforementioned limitations to data-driven models and biomass optimization-based strategies advocate the need for a computational tool which can compute biochemically relevant parameters directly from a modeled network. This implies that the parameter should be derivable, measurable and its analysis should be able to generate testable hypotheses. The dissociation constant is an empirically determined parameter, which can be mapped to several biochemically relevant outcomes of a reaction (forward, reverse, equivalent, and tight binding) (Furukawa et al., 2016; Yu and Craciun, 2018; Gerstl et al., 2019; Sparks et al., 2019; Kundu, 2022; Sura and Antalik, 2022). The probable dissociation constant for a reaction is a numerical measure that is computed from a null space-generated subspace of the stoichiometry number matrix for a biochemical network and possesses several desirable properties of the true dissociation constant (Kundu, 2023a). Here, we present “ReDirection,” an R-package which can compute the probable dissociation constant for every reaction of a user-defined biochemical network (Kundu, 2023b). This paper introduces some of the principles and definitions used by “ReDirection” to compute the probable dissociation constant for a user-defined biochemical network. An outline of the functions used by “ReDirection,” their dependencies, rationale, and usage is presented. A stepwise description and brief analysis of the algorithm that “ReDirection” deploys are also described, followed by numerical studies on constrained biochemical networks of human galactose metabolism and heme and cholesterol biosynthesis. The paper concludes with a summary of the salient features, limitations, and future studies which may utilize “ReDirection.”

## 2 Methods

### 2.1 Definitions, preliminary concepts, and notations relevant to comprehending the functionality of “ReDirection”

The algorithm deployed by “ReDirection” is mathematically rigorous and biochemically relevant, and has been extensively discussed (Kundu, 2023a; Kundu, 2023b). Briefly, a biochemical network is modeled as the sparse stoichiometry number matrix $\left(p∼S\left(p\right)\subset Z^{J\times \overset{═}{I}}\right)$ and is a collection of i -indexed $\left(i=1,2\ldots \overset{═}{I}\right)$ r -reaction vectors $\left(r_{i}\in Z^{j}\right)$ (Def. (1a)) (Kundu, 2023a; Kundu, 2023b). Each reaction vector is populated by combinations of j -indexed $\left(j=1,2\ldots J\right)$ m -stoichiometry numbers $\left(m_{j}\in Z\right)$ of J -reactants/products (Def. (1b)) (Kundu, 2023a; Kundu, 2023b). The modeled biochemical network is subsumed to operate under several biochemically relevant numerical constraints. These include lower bounds for the numbers of reactants/products and reaction vectors, modeling a reaction vector as the interaction between one or more pairs of molecules (enzymatic or non-enzymatic) with differently signed stoichiometry numbers, equilibrium $\left(S≃0\right),$ and fixed outcomes for a participating reaction (forward, reverse, and equivalent) (Def. (2)) (Kundu, 2023a; Kundu, 2023b).

“ReDirection” is assessed by the time needed $\left(T\;\min\right)$ to unambiguously assign an outcome to every reaction (Def. (3)). This depends on the architecture and complexity of the numerical values that constitute the stoichiometric number matrix of the modeled biochemical network, the nullity of the null space, and a network-suitable null space-generated subspace (Kundu, 2023a; Kundu, 2023b). For a stoichiometry number matrix, the desired null space is

$$\left\{v_{k}\in V{\subset R}^{\overset{═}{I}}|{v_{k}}^{T}\times S_{p}={\vec{0}}^{T}\right\},$$ (Def. 4)

where

$$V≝Null\;space\;of\;S_{p},$$ (1)

#V≥2, (2)

k=1,2…K, (3)

K=#V, (4)

$$S\left(p\right)\subset Z^{J\times \overset{═}{I}}.$$ (5)

“ReDirection” combinatorially sums the vectors of the null space and, thence, each null space-generated subspace (Kundu, 2023a). This results in several subsets of vectors which contribute to the cardinality of each null space-generated subspace and may be summarized. We describe this comprehensive null space-generated subspace as the set which contains trivial vectors, along with redundant or finite occurrences of non-trivial and identical null space vectors (Kundu, 2023a):

$$\overset{═}{V}=V\cup \overset{═}{H}\cup H\cup L,$$ (Def. 5)

where

V≝Null space of user−defined stichiometry number matrix,

L≝Set of trivial null space vectors, (Def. 6)

H≝Set of unique null space vectors, (Def. 7)

$$\overset{═}{H}≝Set\;of\;vectors\;which\;have\;one\;or\;more\;subsets\;of\;identical\;vectors.$$ (Def. 8)

Rewriting **Def. (4)** to include these vectors yields

$$\left\{v_{k}\in \overset{═}{V}\subseteq V{\subset R}^{\overset{═}{I}}|{v_{k}}^{T}\times S_{p}={\vec{0}}^{T}\right\},$$ (Def. 9)

where

$$\overset{═}{V}≝Comprehensive\;subspace\;of\;V\;with\;cardinality\;\#\overset{═}{V},$$

$$V≝Null\;space\;of\;S_{p},$$

$$v_{k}=\vec{0}\;\left(Trivial\;null\;space\;vector\right),$$ (6)

$$v_{k}\neq \vec{0}\;\left(Non-trivial\;null\;space\;vector\right),$$ (7)

k=1,2…K,

$$K=\#\overset{═}{V.}$$

We now enumerate various cases that may arise when we combinatorially sum non-trivial vectors:

$$\begin{array}{rrr} Case\;1: & If\left(v_{1}\neq v_{2}\ldots \neq v_{K}\neq 0\right), & \\ & then,\left(\left\{v_{1},v_{2}\ldots ,v_{K}\right\}\in H\subset \overset{═}{V}\right)\text{ and }\left(\overset{═}{H}=\emptyset \right), & \mathrm{Def}.\left(10\right)\\ \Rightarrow & \#H=K;\#\overset{═}{H}=0. & \left(8\right),\left(9\right)\\ Case\;2a: & Let\left(\begin{array}{c} v_{1}=v_{2}\ldots =v_{K}\neq 0\\ where\;\left\{v_{1},v_{2}\ldots ,v_{K-1}\right\}\in \overset{═}{H} \end{array}\right), & \mathrm{Def}.\left(11\right)\\ Case\;2b: & If\left(\begin{array}{c} \left(v_{1}=v_{2}\ldots =v_{A}\right),\left(v_{A+1}=v_{A+2}\ldots =v_{B}\right)\ldots \left(v_{K-\left(K+1\right)}\ldots =v_{K-1}=v_{K}\right)\\ where\;\left\{v_{1},v_{2}\ldots ,v_{K}\right\}\in \overset{═}{H} \end{array}\right), & \mathrm{Def}.\;\left(12\right) \end{array}$$

We can immediately see from Case 2 that it is possible to have a finite number of subsets of non-trivial identical vectors exist in H═ which is dependent on its cardinality (Kundu, 2023a). We will formally define the number of subsets that can be formed as $\left(\tau \right)$ (**Def. (13**)) (Kundu, 2023a),

$$\begin{array}{rr} \tau ≝1+\sum_{t=2}^{t=\#\overset{═}{H}-2}\left(\begin{array}{c} \#\overset{═}{H}\\ t \end{array}\right), & \left(10\right)\\ =\left\{\begin{array}{c} 1\;iff\;\overset{═}{H}=\left\{2,3\right\}\\ \geq 2\;iff\;\overset{═}{H}\geq 4 \end{array}\right.. & \left(10.1\right) \end{array}$$

We define the exact number of vectors to be reassigned on account of their uniqueness as τ═ , i.e., from H═ to H (**Def. (14)**). We will now compute the number finite subsets for different cardinalities of H═ ,

For τ=1 ,

$$\begin{array}{rrr} & if\left(v_{1}=v_{2}\ldots =v_{K}|K=\#\overset{═}{H}=\left\{2,3\right\}\right), & \\ & then,\left(\begin{array}{c} v_{1}\vee v_{2}\ldots \vee v_{K}={⋁}_{k=1}^{k=K}v_{k}\in H\\ and,\\ \left\{v_{1},v_{2}\ldots ,v_{K-1}\right\}\in \overset{═}{H} \end{array}\right), & \mathrm{Def}.\left(15\right) \end{array}$$

$$\begin{array}{cc} Clearly, & \overset{═}{\tau }=1 \end{array},$$ (11)

$$\tau \begin{array}{cc} \Rightarrow \#H & =\#H+\overset{═}{\tau ,} \end{array}$$ (12)

=#H+1, (12.1)

$$\begin{array}{cc} and,\#\overset{═}{H} & =A-1, \end{array}$$ (13)

$$=\#\overset{═}{H}-1.$$ (13.1)

For τ=2 , the corresponding data is

$$if\;\left(v_{1}=v_{2}\ldots =v_{A}\right)\;and\;\left(v_{A+1}=v_{A+2}\ldots =v_{K}\right)|K=\#\overset{═}{H}\geq 4,$$

$$then,\left(\begin{array}{c} \left(\begin{array}{c} v_{1}\vee v_{2}\ldots \vee v_{A}={⋁}_{k=1}^{k=A}v_{k}\in H\\ and,\\ \left\{v_{1},v_{2}\ldots ,v_{A-1}\right\}\in \overset{═}{H} \end{array}\right)\;and\\ \left(\begin{array}{c} v_{A+1}\vee v_{A+2}\ldots \vee v_{K}={⋁}_{k=A+1}^{k=K}v_{k}\in H\\ and,\\ \left\{v_{A+1}\vee v_{A+2}\ldots ,v_{K-A-1}\right\}\in \overset{═}{H} \end{array}\right) \end{array}\right).$$ (Def. 16)

$$\begin{array}{cc} Here, & {\overset{═}{\tau }}_{A}={\overset{═}{\tau }}_{K}=1 \end{array},$$ (14)

$$\begin{array}{cc} \Rightarrow & \overset{═}{\tau }∼\tau_{A}+\tau_{K}, \end{array}$$ (15)

=2, (15.1)

$$\begin{array}{cc} \Rightarrow \#H & =\#H+\tau \end{array},$$ (16)

=#H+2, (16.1)

$$\begin{array}{cc} and\;\#\overset{═}{H} & =\left(A-1\right)+\left(K-A-1\right), \end{array}$$ (17)

=K−2, (17.1)

$$=\#\overset{═}{H}-2.$$ (17.2)

In general, for τ-subsets of identical vectors in $\overset{═}{H}$ the number of $\overset{═}{\tau }$ -vectors that will be reassigned will be numerically identical ( $\tau ∼\overset{═}{\tau }$ ),

$$\#H=\#H+\overset{═}{\tau },$$ (18)

$$\#\overset{═}{H}=\#\overset{═}{H}-\overset{═}{\tau },$$ (19)

### 2.2 Generic description, availability, and guidelines for using “ReDirection”

**FIGURE 1:**
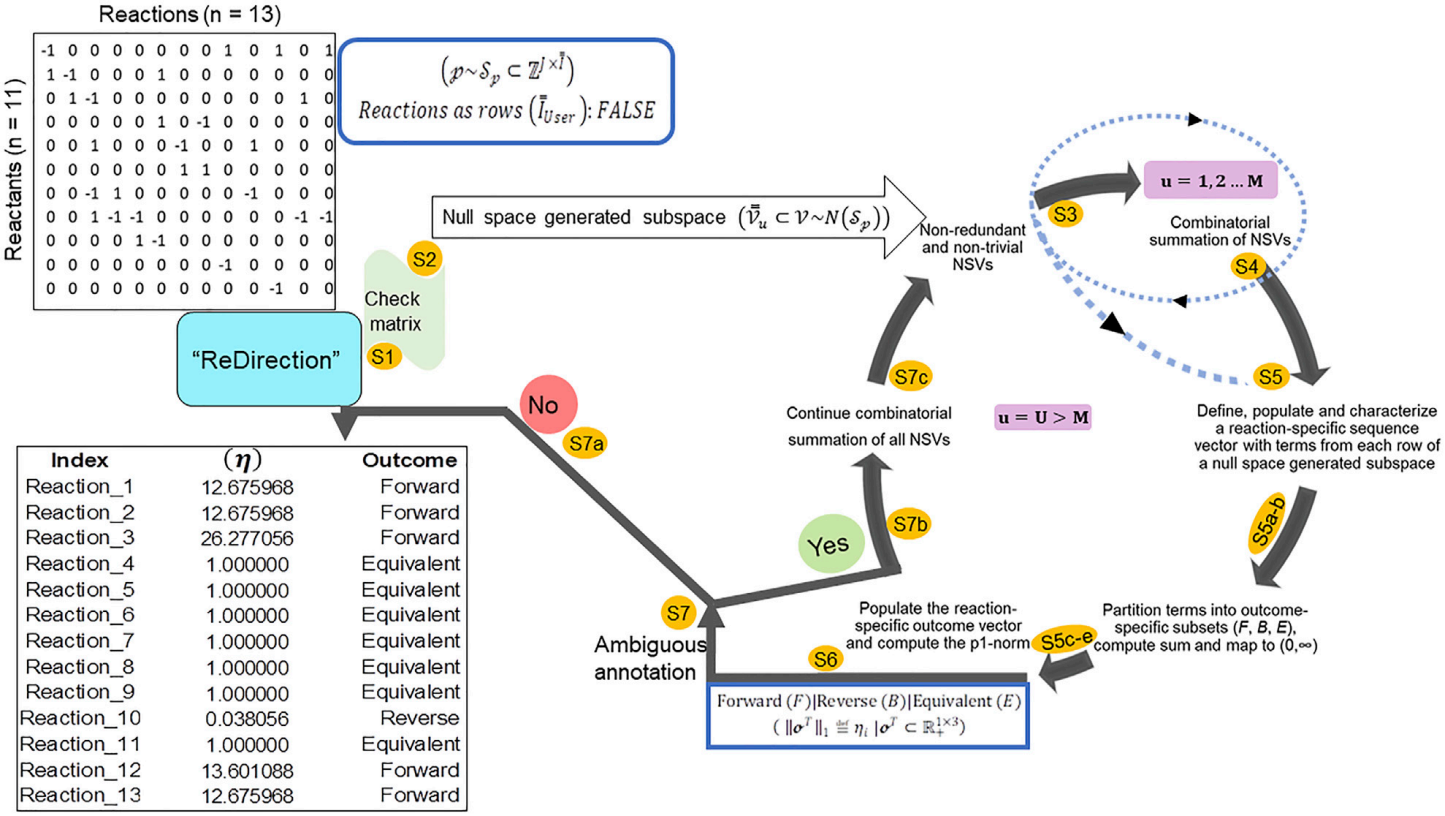
Schematic representation of the steps deployed by “ReDirection” to characterize every reaction of a user-defined biochemical network with the probable dissociation constant: “ReDirection” checks the stoichiometry number matrix that is provided by the user for a modeled biochemical network for compliance with pre-defined criteria. If true, then “ReDirection” computes a null space-generated subspace by excluding all redundant and trivial vectors, and combinatorially summing the vectors that remain. “ReDirection” also defines a reaction-specific sequence vector which comprises terms drawn from each row of the resulting subspace. “ReDirection” computes several descriptors (mathematical, statistical) for the numerical values that comprise this vector and partitions these into distinct subsets in accordance with the expected outcomes (forward, reverse, and equivalent) for a reaction. “ReDirection” then maps the sum of the terms of each outcome-specific subset to the strictly positive real number and bins these to a reaction-specific outcome vector. The p1-norm of this vector is the probable dissociation constant for a reaction and is used to annotate the same. “ReDirection” accomplishes this recursively and over several iterations until every reaction of the modeled biochemical network has been assigned an unambiguous outcome. Abbreviations: $\eta_{i}$ , probable dissociation constant for the $i^{th}$ -reaction of a user-defined biochemical network; $S_{p}$ , user-defined stoichiometry number matrix for a biochemical network; S1-7, steps of the algorithm deployed by “ReDirection” to compute the probable dissociation constant and assign an outcome to every reaction of a user-defined biochemical network; NSV, null space-generated subspace vector.

“ReDirection” is freely available and can be updated or installed directly from the graphics user interface (GUI) (R-4.1. x) as “update.packages (‘ReDirection’)” and/or “install.packages (‘ReDirection’)” from any of the CRAN mirrors. “ReDirection” is built in RStudio (1.4.1717) and tested in R-4.1. x. “ReDirection” comprises three functions (*calculate_reaction_vector*, *check_matrix*, and *reaction_vector*). The dependencies for “ReDirection” are the packages “pracma,” “MASS,” “stats,” and the *combinations* function from the R-package (“gtools”). The downloaded package includes detailed documentation of all the functions, along with ready-to-use examples and tests of functionality. “ReDirection” utilizes these functions sequentially and processes the stoichiometry number matrix of the reactants/products and reactions of a biochemical network that is defined by the user (Figure 1). In addition to implementing “ReDirection” locally, several R-scripts are developed in house, and used to preformat (input and output) and analyze data. The algorithm followed by “ReDirection” can be divided into simpler steps. These include checking the user-defined stoichiometry matrix, searching for a suitable null space-generated subspace, screening and partitioning terms, and computing the probable dissociation constant (Figure 1).

#### 2.2.1 Checking the user-defined stoichiometry number matrix for a biochemical network

Although “ReDirection” is simple to operate, there are a few guidelines that the user needs to be aware of whilst using it. “ReDirection” is reaction-centric and requires that the number of reactions and reactants/products of a modeled biochemical network strictly conforms to the lower bounds for each (Kundu, 2023a). Since the user is not expected to validate the stoichiometry number matrix manually, “ReDirection” undertakes this task and carries out this unequivocally prior to commencing the iterations. In addition to the stoichiometry number matrix, the user is expected to provide a logical argument (TRUE, FALSE) that indicates whether the reactions are to be considered rows or columns,

$$\begin{array}{cc} TRUE & ∶=Reactions\;as\;rows\left(S_{p}\right), \end{array}$$ (Def. 17)

$$\begin{array}{cc} FALSE & ∶=Reactions\;as\;columns\left(S_{p}\right) \end{array}.$$ (Def. 18)

$$S_{p}∼\left\{\begin{array}{c} S_{p}\;iff\;TRUE,\\ {S_{p}}^{T}\;if\;FALSE. \end{array}\right.$$ (Def. 19)

“ReDirection” utilizes these data to assign the appropriate orientation to the stoichiometry number matrix (step 1; Figure 1),

Another checkpoint, albeit internal, is the identification and subsequent exclusion of linear dependent row and column vectors that are contributed by half-reactions (forward, reverse) of the modeled biochemical network (step 1; Figure 1). “ReDirection” accomplishes this by recursively multiplying each reaction vector $\left(r_{i}\in Z^{J}\right)$ with the scalar quantity $\left(-1\right)$ and checking whether this results in a duplicate vector (Kundu, 2023a). If this is true, then “ReDirection” excludes this reaction vector,

$$If\left(\begin{array}{c} r_{x},r_{y}\in Z^{J}\;s.t.\;r_{y}=\left(-1\right).r_{x}\\ where\;x\neq y\;and\;x,y=1,2\ldots \overset{═}{I} \end{array}\right),$$ (Def. 20)

$$then\;\left(\left(-1\right).r_{y}=\left(-1\right).\left(-1\right).r_{x}=r_{x}\;for\;each\;x=1,2\ldots \overset{═}{I}\right)$$ (20,21)

$$and\;r_{x}\vee r_{y}\notin Z^{J}.$$

It is clear that the final list of reactions that “ReDirection” $\left({\overset{═}{I}}_{\prime \prime ReDirection\prime \prime }\right)$ considers is only half of what may have originally been entered by the user, ${\overset{═}{I}}_{p}∼{\overset{═}{I}}_{User}$ ; Eq. (22), for the complete biochemical network,

$$\begin{array}{cc} {\overset{═}{I}}_{\prime \prime ReDirection\prime \prime } & <{\overset{═}{I}}_{p} \end{array},$$ (23)

$$=\frac{{\overset{═}{I}}_{p}}{2},$$ (23.1)

$$∼\frac{{\overset{═}{I}}_{User}}{2}.$$ (23.2)

The modified stoichiometry number matrix is now

$$S_{p}\subset Z^{J\times {\overset{═}{I}}_{\prime \prime ReDirection^{\prime \prime }}}$$ (24)

$$=Z^{J\times \left(\frac{{\overset{═}{I}}_{User}}{2}\right)}.$$ (24.1)

“ReDirection” rechecks the modified stoichiometry number matrix (steps 1–3; Figure 1),

$$\begin{array}{cc} {\overset{═}{I}}_{\prime \prime ReDirection\prime \prime } & \geq J+2, \end{array}$$ (25)

$$\begin{array}{cc} \Rightarrow {\overset{═}{I}}_{\prime \prime ReDirection\prime \prime } & >rank\left(S_{p}\right), \end{array}$$ (26)

$$\begin{array}{cc} {\overset{═}{I}}_{\prime \prime ReDirection\prime \prime } & \geq 6. \end{array}$$ (27)

“ReDirection” rechecks the modified stoichiometry number matrix (steps 1–3; Figure 1),

If there are no further deficiencies, “ReDirection” computes the null space (Step 2; Figure 1):

$$\begin{array}{cc} V & ∼Null\;space\left(S_{p}\right) \end{array},$$ (Def. 21)

$$≝\left\{\begin{array}{c} Null\;space\left(S_{p}\right)\;for\;reactions\;as\;rows\;ofS_{p},\\ Null\;space\left({S_{p}}^{T}\right)\;for\;reactions\;as\;Columns\;of\;S_{p}. \end{array}\right.$$ (28)

#### 2.2.2 “ReDirection”-mediated search for a suitable null space-generated subspace to compute the probable dissociation constant for every reaction of a biochemical network

“ReDirection” then searches for a suitable null space-generated subspace $\left(\overset{═}{V}\subset V\right)$ to compute the probable dissociation constant for every reaction of a user-defined biochemical network. “ReDirection” does this by combinatorially summing only non-trivial and unique null space vectors over several iterations. Let us describe this null space-generated subspace as a function of u -iterations, where u=1,2…U∈N ,

$$\overset{═}{V}∼{\overset{═}{V}}_{u}∋v_{u_{k}}\in R^{\overset{═}{I}},$$ (29)

$${\overset{═}{V}}_{u}≝Comprehensive\;subspace\;of\;V\;for\;the\;u^{th}-iteration,$$ (30)

where

k=1,2…K

u=1,2…U∈N, (31)

$$K=\#{\overset{═}{V}}_{u}.$$

Rewriting ${\overset{═}{V}}_{u}$ in terms of the subsets $H,\overset{═}{H},L$ whilst preserving the null space spanning vectors $\left(V\right)$ , we obtain

$${\overset{═}{V}}_{u}=V\cup H_{u}\cup {{\overset{═}{H}}_{u}\cup L}_{u}.$$ (32)

Clearly, with each iteration, the computational complexity increases with a corresponding increase in the time required by “ReDirection” to completely annotate every reaction of a biochemical network. Therefore, “ReDirection” identifies and excludes these vectors in an attempt to complete the annotations within a reasonable amount of time (steps 3–7; Figure 1). The pseudocode for the case where the nullity of the null space $\left(\#V=2\right)$ is presented and discussed for a null space-generated subspace in terms of the $u^{th}$ -iteration is shown in Table 1.

**TABLE 1:** Pseudocode to determine cardinality as the function of a finite number of u -iterations, for a null space-generated subspace where the nullity for a stoichiometry number matrix is 2

a,b:Null space vectors
u:Number of iterations
A:Cardinality of null space or null space−generated subspace
$\overset{═}{A}:Incremented\;cardinality\;ofnull\;space-generated\;subspace$
t:Combinatorial index
K:Number of summed vectors
τ:Number of groups of summed and identical vectors
$\overset{═}{\tau }:Number\;of\;vectors\;of\;summed\;and\;identical\;vectors\;to\;include$
w:Number of vectors to exclude
u←1
A←2
Start:
$\overset{═}{A},w,\overset{═}{\tau },\tau \leftarrow 0$
t←2…A
$K\leftarrow \sum_{t=2}^{t=A}\left(\begin{array}{c} A\\ t \end{array}\right)$
$if\left(X_{1}=X_{2}\ldots {=X}_{K}=0\right)\;then,$
w←w+K
endif
$elseif\left(X_{1}\neq X_{2}\ldots {\neq X}_{K}\neq 0\right)\;then,$
$\overset{═}{A}\leftarrow A+K$
endif
$elseif\left(X_{1}|=X_{2}\ldots =X_{A}\neq 0\right)\;and\;\left(X_{A+1}=X_{A+2}\ldots =X_{K}\neq 0\right)\;then,$
τ←2
$\overset{═}{\tau }\leftarrow \tau$
$w\leftarrow (w+K)-\overset{═}{\tau }$
$\overset{═}{A}\leftarrow A+\overset{═}{\tau }$
endif
u←u+1
$A\leftarrow \overset{═}{A}$
$\overset{═}{A},w,\overset{═}{\tau },\tau \leftarrow 0$
Next:

#### 2.2.3 Row-wise screening and partitioning of terms of the selected null space-generated subspace

Every row of this $u^{th}$ -iteration-specific and null space-generated subspace is redefined as an $i^{th}$ -reaction-specific sequence vector and is characterized by several numerical descriptors such as the number of terms, mean, standard deviation, and upper and lower bounds (Def. (22)) (Kundu, 2023a). On the basis of these descriptors, the terms from each row are binned to the outcome-specific subsets forward (*F*), reverse (*B*), or equivalent (*E*), summed, and mapped to strictly positive real numbers (Def. (23); Eqs (33–43) (Table 2) (Kundu, 2023a). The mapped terms populate the $i^{th}$ -reaction-specific output vector with a p1-norm, which is the probable dissociation constant for the $t^{th}$ -reaction (Defs. (24, 25); Eqs (44–48)) (Table 2) (Kundu, 2023a). “ReDirection” implements this algorithm iteratively and recursively, and computes the probable dissociation constant for every reaction of a user-defined biochemical network.

**TABLE 2:** “ReDirection”-based computation of the probable dissociation constant for the $i^{th}$ -reaction of a user-defined biochemical network.

Analysis and mapping	Subset (*F*)		Subset (*B*)		Subset (*E*)	
Output-specific sum of terms (domain): $x\left(.\right)\in R\cap \left(-\infty ,\infty \right)$	$\begin{array}{cc} Case\;1: & x_{1}>1\\ Case\;2: & x_{2}\in \left(0,1\right) \end{array}$	(33) (34)	$\begin{array}{cc} Case\;1: & -x_{2}\in \left(-1,0\right)\\ Case\;2: & x_{3}<-1 \end{array}$	(37) (38)	$\left|x_{4}\right|\approx 0$	(41)
Linear map: $g:x\in R\cap \left(-\infty ,\infty \right)\mapsto y\in R\cap \left(0,\infty \right)$	$\begin{array}{rr} y_{F} & ≝g\left(x_{1}\right)\;or\;g\left(x_{2}\right)\\ & =x_{1}\;or\;x_{2} \end{array}$	(35) (35.1)	$\begin{array}{rr} y_{B} & ≝g\left(-x_{2}\right)\;or\;g\left(x_{3}\right)\\ & =e^{-x_{2}}\;or\;e^{x_{3}} \end{array}$	(39) (39.1)	$\begin{array}{rr} y_{E} & ≝g\left(x_{4}\right)\\ & =e^{x_{4}} \end{array}$	(42) (42.1)
Range: $y\left(.\right)\in R\cap \left(0,\infty \right)$	$y_{F}\in R\cap \left(0,\infty \right)$	(36)	$y_{B}\in R\cap \left(0,\infty \right)$	(40)	$y_{E}\in R\cap \left\{1\right\}$	(43)
Reaction-specific outcome vector: $o^{T}$	$\begin{array}{rrr} {\left[\left(g\left(x_{1}\right)\;or\;g\left(x_{2}\right)\right)\;\left(g\left(-x_{2}\right)\;or\;g\left(x_{3}\right)\right)\;\left(g\left(x_{4}\right)\right)\right]}^{T} & ={\left[\left(x_{1}\;or\;x_{2}\right)\;\left(e^{{-x}_{2}}\;or\;e^{x_{3}}\right)\;\left(e^{x_{4}}\right)\right]}^{T} & \left(44\right)\\ & ={\left[y_{F}\;y_{B}\;y_{E}\right]}^{T} & \left(44.1\right) \end{array}$					
Prediction	Forward		Reverse		Equivalent	
Probable dissociation constant (p1-norm): $\begin{array}{cc} {\left\|o^{T}\right\|}_{1} & =\eta_{i}\in R\cap \left(0,\infty \right) \end{array}$	$\begin{array}{rr} Case\;1: & x_{1}+0+0\\ & =\eta_{i}\in R\cap \left(1,\infty \right)\\ Case\;2: & x_{2}+e^{-x_{2}}+0\\ & =\eta_{i}\in R\cap \left(1,\infty \right) \end{array}$	(45) (45.1) (46) (46.1)	$\begin{array}{l} 0+e^{x_{3}}+0\\ {=\eta }_{i}\in R\cap \left(0,1\right) \end{array}$	(47) (47.1)	$\begin{array}{l} 0+0+e^{x_{4}}\\ {=\eta }_{i}\in R\cap \left\{1\right\} \end{array}$	(48) (48.1)

Abbreviations: $x\left(.\right)$ , real-valued numeral of a null space-generated subspace; $g:x\left(.\right)$ , linear map for a real-valued numeral of a null space-generated subspace; $y\left(.\right)$ , strictly positive mapped real-valued numeral; $o^{T}$ , reaction-specific outcome vector; i , $i^{th}$ -reaction of a user-defined biochemical network; $\eta_{i}$ , probable dissociation constant for the $i^{th}$ -reaction of a user-defined biochemical network; F,B,E , outcome-specific subsets (forward, *F*; reverse, *B*; and equivalent, *E*).

### 2.3 “ReDirection”-based numerical studies to ascertain and assess an upper bound for the maximum number of reactions for a user-defined biochemical network

It has already been proven that the algorithm deployed by “ReDirection” is likely to be NP-hard (Kundu, 2023a). This means that for a biochemical network whose output is determined by summing its constituent terms, there is a limit on the maximum number of reaction vectors that can be modeled by a user. Since “ReDirection” utilizes combinatorial summations to identify a suitable null space-generated subspace from where the probable reaction constants for a modeled biochemical network can be computed, the upper bound for the maximum number of reaction vectors is likely to be lower, i.e., there is a narrow permissible limit.

**FIGURE 2:**
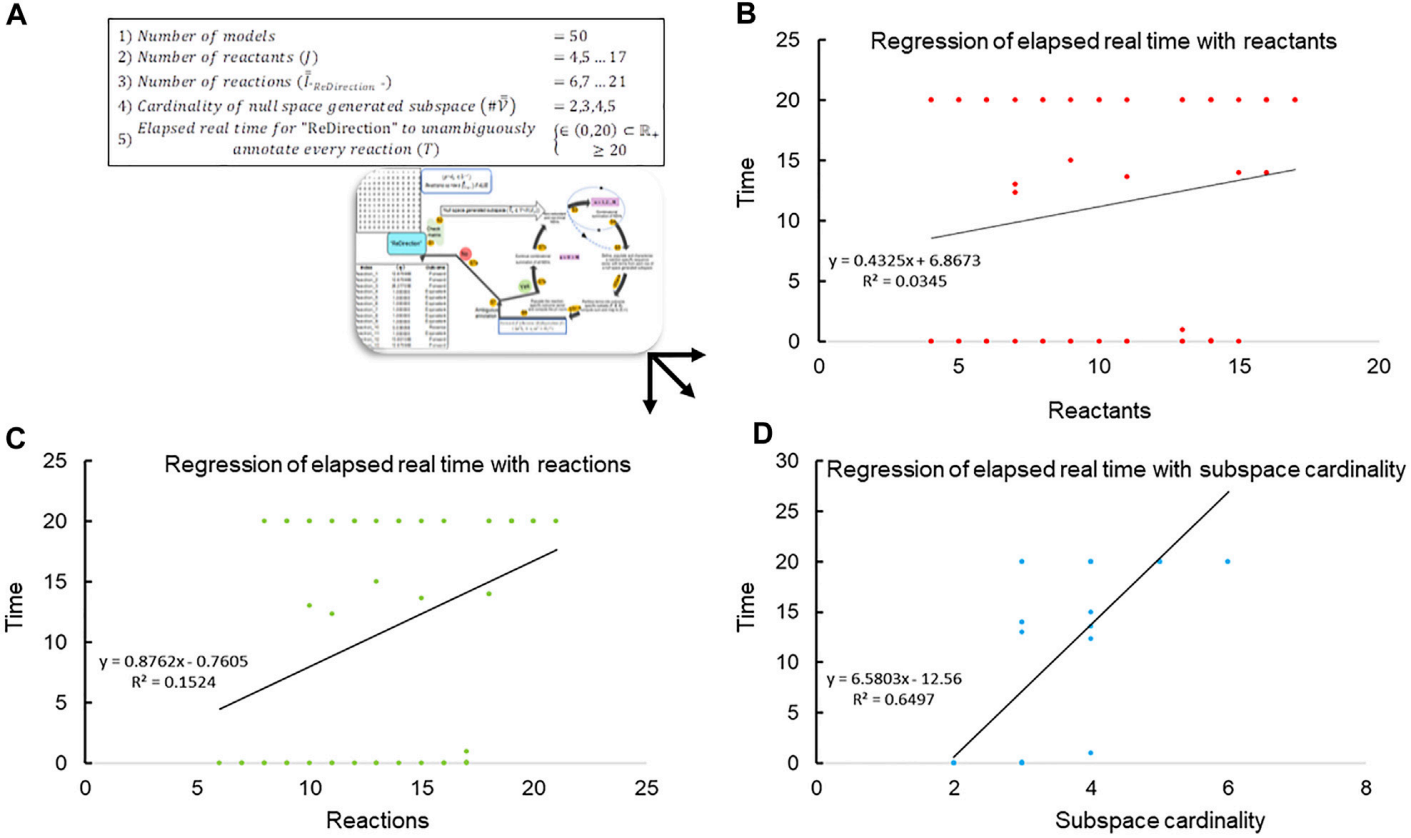
Regression of elapsed real time with network-specific parameters. **(A)** The data, i.e., elapsed run-time ( min ) from several observations ( n=50 ), are plotted against the network-specific parameters of reactant number, reaction number, and the cardinality of the reaction-specific null space-generated subspace chosen by “ReDirection” to compute the probable dissociation constant for a reaction. The scatter plot data are modeled with a specific linear regression equation, and the relevant coefficient of differentiation ( $R^{2}$ ) is highlighted; **(B)** scatter diagram of the run time that elapses when “ReDirection” attempts to unambiguously annotate every reaction of a simulated biochemical network with the number of reactants/products that participate in the network; **(C)** scatter diagram of the run time that elapses when “ReDirection” attempts to unambiguously annotate every reaction of a simulated biochemical network with the number of reactions that participate in the network; and **(D)** scatter diagram of the run time that elapses when “ReDirection” attempts to unambiguously annotate every reaction of a simulated biochemical network with the cardinality of a reaction-specific null space-generated subspace that is chosen by “ReDirection” to compute the probable dissociation constant for a reaction.

Since “ReDirection” needs to be user-friendly, an indicator of this must be available *a priori*. We utilize the time metric to ascertain this numerically. In other words, the time $\left(T\;\min\right)$ that “ReDirection” takes to unambiguously annotate every reaction of a biochemical network is utilized to delineate an upper bound for the maximum number of reaction vectors that the user can incorporate for a biochemical network. We create several stoichiometry number matrices $\left(n=50\right)$ in accordance with the previously established constraints and examine the run-time that “ReDirection” takes to compute the probable dissociation constants for the simulated yet plausible biochemical networks (Figure 2A; Supplementary Text S1) (Kundu, 2023a). The studies are carried out on a system with the following configuration: i5-10400F processor, clock speed 2.9 GHz, 64-bit, 16 GB RAM.

In order to assess these observations, we compute a truth table with the following assumptions and abbreviations (Defs 26–29):

$$\begin{array}{rr} TP & ∶=Time\;\left(T\right)-to-unambiguous\;annotation\;of\;reaction\;where\;\#V\in \left[2,4\right]\;and\;T\in \left(0\;\min,20\;\min\right),\\ FP & ∶=Time\;\left(T\right)-to-unambiguous\;annotation\;of\;reaction\;where\;\#V>4\;and\;T\in \left(0\;\min,20\;\min\right),\\ FN & ∶=Time\;\left(T\right)-to-unambiguous\;annotation\;of\;reaction\;where\;\#V\in \left[2,4\right]\;and\;T>20\;\min,\\ TN & ∶=Time\;\left(T\right)-to-unambiguous\;annotation\;of\;reaction\;where\;\#V>4\;and\;T>20\;\min. \end{array}$$

This yields the following indices to assess our premise:

$$\begin{array}{cc} Precision & ≝\frac{TP}{TP+FP}, \end{array}$$ (49)

$$\begin{array}{cc} Recall\;\left(sensitivity\right) & ≝\frac{TP}{TP+FN}, \end{array}$$ (50)

$$\begin{array}{cc} Specificity & ≝1-\frac{FP}{FP+TN}, \end{array}$$ (51)

$$=\frac{TN}{TN+FN},$$ (51.1)

$$\begin{array}{cc} Accuracy & ≝\frac{TP+TN}{TP+FP+FN+TN}. \end{array}$$ (52)

### 2.4 “ReDirection”-based studies on physiologically relevant biochemical networks

**FIGURE 3:**
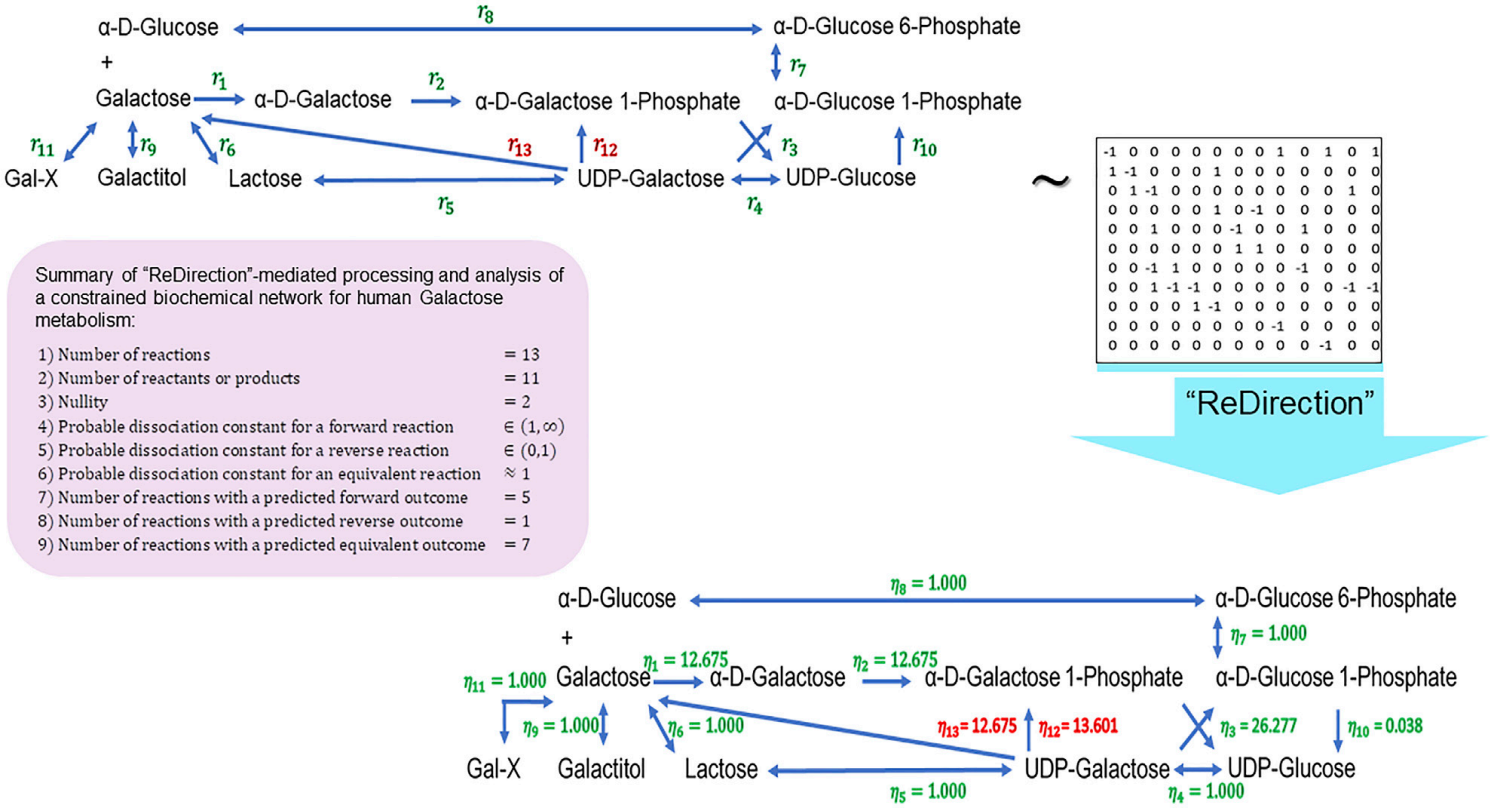
Schematic representation of a “ReDirection”-mediated investigation of a constrained biochemical network for human galactose metabolism. The biochemical network for galactose metabolism in *Homo sapiens* comprises several potentially bidirectional reactions. Here, “ReDirection” investigates the conversion of UDP-galactose to alpha-D-galactose 1-phosphate ( $r_{12}$ ) and D-galactose ( $r_{13}$ ) via alternate pathways on the unperturbed set of reactions $\left(r_{1}-r_{11}\right)$ . The data suggest that a large proportion of the reactions is equivalent and may, therefore, function to regulate galactose metabolism. Additionally, the net direction that is observed before and after perturbing the system is toward the biosynthesis of UDP-glucose. This is in accordance with the relatively milder clinical manifestations of inborn errors of metabolism that arise due to mutations in the enzymes (epimerase, kinase) of the pathway. Abbreviations: $\eta_{i}$ , probable dissociation constant for the $i^{th}$ -reaction of a constrained biochemical network of human galactose metabolism; Gal-X, galactose containing di (galactinol, melibitol, epimelibiose)- or oligo (stachyose)-saccharides, which are cleaved by beta-galactosidase ( EC 3.2.1.22 ); UDP, uridine-di-phosphate; UTP, uridine tri-phosphate.

**FIGURE 4:**
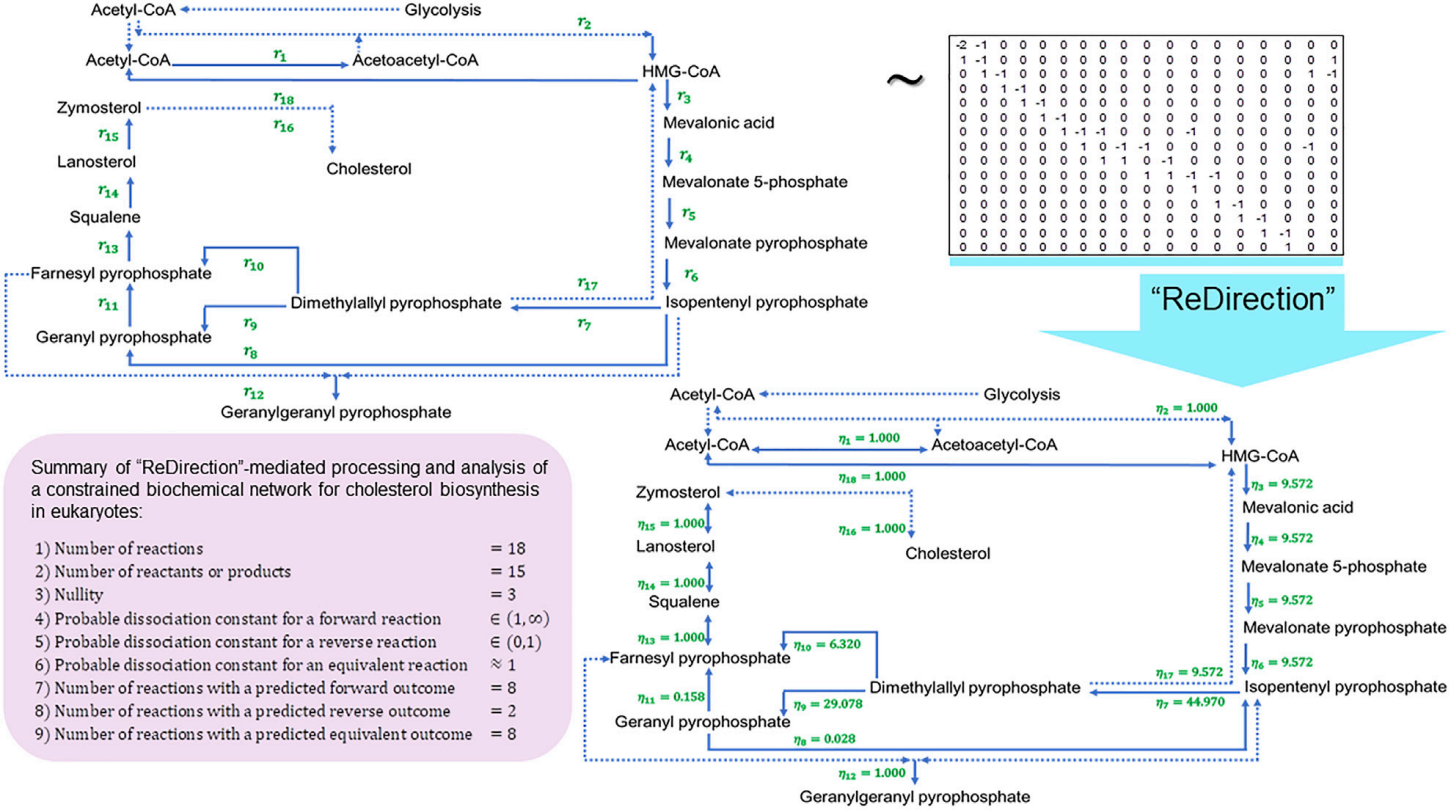
Schematic representation of a “ReDirection”-mediated investigation of a constrained biochemical network for eukaryotic cholesterol biosynthesis. The high number of predicted equivalent reactions $\left(\approx 44\%\right)$ for cholesterol biosynthesis suggests a regulatory role and may, therefore, be a reason why this pathway is conserved across eukaryotes, bacteria, and archaea. The shunt pathway is a simple yet effective way of redirecting mevalonate prior to ring closure. Smith–Lemli–Opitz syndrome is an inborn error of metabolism that arises due to mutations in the terminal enzyme of cholesterol biosynthesis (delta-7-reductase; EC 1.3.1.21 ) and is postulated to cause an increased flux of mevalonate through the shunt pathway, along with a concomitant increase in the excretion of urinary mevalonate. The results of this study support this notion with all the probable dissociation constants favoring a prominent role for the shunt pathway. The isomeric conversion of isopentenyl pyrophosphate to dimethylallyl pyrophosphate from mevalonate has the greatest numerical value of all the predicted probable dissociation constants $\left(\eta_{7}\approx 45\right)$ , which also supports the rapid removal of mevalonate either by conversion (main, shunt) and/or excretion in urine. Abbreviations: $\eta_{i}$ , probable dissociation constant for the $i^{th}$ -reaction of a constrained biochemical network for cholesterol biosynthesis.

**FIGURE 5:**
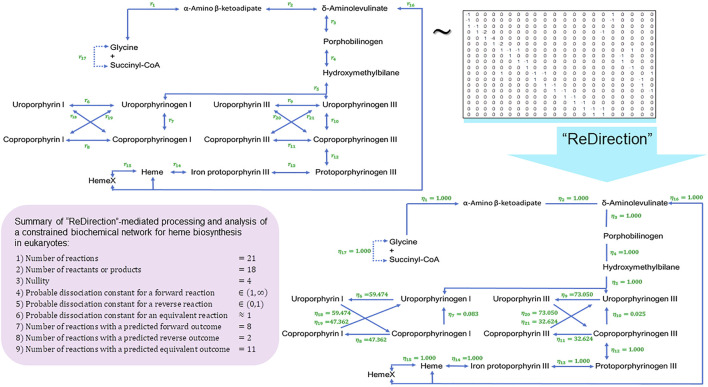
Schematic representation of a “ReDirection”-mediated investigation of a constrained biochemical network for heme biosynthesis. Here, we present a biochemical network which examines the effects of the uroporphyrins (I) and (III) and coproporphyrins (I) and (III) on the immediate precursors uroporphyrinogens (I) and (III) or products coproporphyrinogens (I) and (III) on the flux of heme $\left(\eta_{6},\eta_{7},\eta_{8},\eta_{18},\eta_{19};\eta_{9},\eta_{10},\eta_{11},\eta_{20},\eta_{21}\right)$ . The uroporphyrins (I) and (III) and coproporphyrins (I) and (III) are generated by sunlight or the spontaneous removal of protons and can function as organic free radicals. Here, we examine the premise that once generated; the free radical cycle involving these is self-propagating and can considerably damage the neighboring skin and other tissues. Interestingly, our data $\left(\eta_{7}\approx \eta_{10}\approx 0.03\right)$ offer a plausible explanation into the pathophysiology of porphyria cutanea tarda (PCT). This inborn error of metabolism is due to a defect in the enzyme uroporphyrinogen decarboxylase and results in debilitating blisters on the skin due to exposure to sunlight. Additionally, in the absence of enzyme-catalyzed reactions, the sequestration of the substrates uroporphyrinogens (I) and (III) and/or coproporphyrinogens (I) and (III) ensures, by the law of mass action, that the flux is toward the biosynthesis of heme and its subsequent incorporation into several heme proteins of physiological and biochemical relevance. Abbreviations: $\eta_{i}$ , probable dissociation constant for the $i^{th}$ -reaction of a constrained biochemical network for heme biosynthesis; HemeX, heme-containing proteins.

We conclude this study by examining the relevance of the probable dissociation constants that are computed by “ReDirection” in physiologically relevant biochemical networks for galactose metabolism and heme and cholesterol biosynthesis. The stoichiometry number matrices for these networks are constructed in accordance with the numerical constraints discussed here and in previous work (Figure 3; Figure 4; Figure 5; Supplementary Texts S2–S4) (Kundu, 2023a; Kundu, 2023b).

Galactose–glucose interconversion is readily observed within the cell, catalyzed by the enzyme UDP-galactose 4-epimerase $\left(EC\;5.1.3.2\right)$ , and suggests a biochemical network with several potentially bidirectional reactions (Conte et al., 2021; Nicoli et al., 2021). Here, “ReDirection” computes the probable dissociation constants for every reaction of a constrained biochemical network $\left({\overset{═}{I}}_{Gal}=13,J_{Gal}=11\right)$ for human galactose metabolism (Eqs. (53, 54)) (Figure 3; Supplementary Text S2). The effect of perturbing $r_{1}-r_{11}$ is investigated by introducing the atypical reactions $r_{12}$ (UDP-galactose → alpha-D-galactose 1-phosphate) and $r_{13}$ (UDP-galactose → alpha-D-galactose 1-phosphate) into the network (Figure 3). The enzymes (UTP-hexose 1-phosphate uridyltransferase, EC 2.7.7.10 ; UTP-monosaccharide-1-phosphate uridyltransferase, EC 2.7.7.64 ) that mediate the transformation of UDP-galactose to alpha-D-galactose 1-phosphate are not significant contributors to human galactose metabolism. This reaction is mediated by UDP-glucose-hexose-1-phosphate uridyltransferase ( EC 2.7.7.12 ) ( $r_{9}$ ) and is a major regulatory checkpoint for galactose–glucose interconversion (Figure 3). Cholesterol biosynthesis is the result of the mevalonate and non-mevalonate pathways, along with a well-characterized mitochondrial shunt pathway that may function to protect hydroxy-methyl-glutaryl (HMG) CoA reductase $\left(EC\;1.1.1.34\right)$ from the deleterious effects of mevalonate (Edmond and Popjak, 1974; Nakanishi et al., 1988; Eisenreich et al., 2004; Buhaescu and Izzedine, 2007). Here, we present, analyze, and discuss a biochemical network $\left({\overset{═}{I}}_{Choles}=18,J_{Choles}=15\right)$ for eukaryotic cholesterol synthesis by the mevalonate pathway, along with the shunt pathway (Eqs. (55, 56)) (Figure 4; Supplementary Text S3). Heme biosynthesis is central to the utilization of iron in the transport of oxygen and carbon dioxide via hemoglobin and other proteins, bilirubin-mediated conjugation and excretion of xenobiotics, and electron transfer in oxidative phosphorylation (Paoli et al., 2002; Thom et al., 2013; Poulos, 2014). We present, analyze, and discuss a biochemical network $\left({\overset{═}{I}}_{Heme}=21,J_{Heme}=18\right)$ for heme biosynthesis and explore the effects of uroporphyrins (I) and (III) and coproporphyrins (I) and (III) on the immediate precursors uroporphyrinogens (I) and (III) or products coproporphyrinogens (I) and (III) (Eqs. (57, 58)) (Figure 5; Supplementary Text S4).

## 3 Results and discussion

### 3.1 Steps deployed by “ReDirection” to compute the probable dissociation constant for every reaction of a user-defined biochemical network

“ReDirection” utilizes the aforementioned functions sequentially and processes the stoichiometric number matrix for the biochemical network that is defined by the user and computes the probable dissociation constant for every reaction (Figure 1). This is conducted sequentially as follows:

Step 1 “ReDirection” checks whether the matrix of stoichiometry numbers that the user inputs is compliant with previously outlined criteria and does not have any linear dependent vectors. If found, “ReDirection” excludes them. The modified input matrix is rechecked.

Step 2 “ReDirection” then computes the null space of the checked/rechecked stoichiometric number matrix of the reactants/products and reactions of the user-defined biochemical network.

Step 3 “ReDirection” processes and screens this null space for redundant and/or trivial vectors and defines a subspace by excluding the same.

Step 4 “ReDirection” combinatorially sums the remaining vectors, i.e., non-redundant and non-trivial, and repeats step 3 for a finite number of u -iterations, where u=M∈N .

Step 5 For u=U>M iterations, “ReDirection” defines, populates, and computes several descriptors (sum, arithmetic mean, and standard deviation) for a reaction-specific sequence vector with terms that are drawn from each row of a null space-generated subspace.

Step 5a “ReDirection” tests each term of an $i^{th}$ -reaction-specific sequence vector for convergence.

Step 5b If this term diverges and possesses a numerical value greater than 2 standard deviations from the mean, then this term is binned into the appropriate outcome-specific (forward/reverse/equivalent) subset.

Step 5c The terms of each outcome-specific subset form a finite series whose sum is computed by “ReDirection.”

Step 5d “ReDirection” then maps these sums to strictly positive real numbers which are then specific for each outcome-specific subset.

Step 5e These outcome-specific numerical measures form the $i^{th}$ -reaction-specific outcome vector.

Step 6 “ReDirection” computes the p1-norm of the reaction-specific outcome vector and annotates the reaction.

Step 7 “ReDirection” checks whether the annotations for all the other reactions of the user-defined biochemical network are unambiguous.

Step 7a If there is no reaction that has been annotated ambiguously, then “ReDirection” outputs the predicted outcomes for every reaction of the user-defined biochemical network.

Step 7b If there is a reaction that has been annotated ambiguously, then “ReDirection” continues the iterations.

Step 7c “ReDirection” combinatorially sums all non-redundant and non-trivial null space-generated subspace vectors that remain, defines a new subspace, and repeats steps 5–7.

### 3.2 “ReDirection”-based delineation of an upper bound for the number of reactions of a biochemical network

The data suggest that the cardinality of the null space-generated subspace that is chosen to compute the probable dissociation constant for a reaction determines not only the time taken to complete the computations but also whether this can be accomplished in real time (Figures 2B–D; Table 3). It was observed that this was achievable, i.e., $T\in \left(0\;\min,20\;\min\right),$ regularly for null spaces with 2-, 3-, and 4-null space spanning vectors (Figures 2B–D; Table 3). However, when the nullity exceeded 4, the computations did not terminate even when the run time significantly exceeded 20 min $\left(T>60\;\min\right)$ (Table 2):

$$\begin{array}{cc} T\in \left(0\;\min,20\;\min\right) & iff\;\left(\begin{array}{c} {\overset{═}{I}}_{\prime \prime ReDirection\prime \prime }\in \left[J+2,J+4\right]\\ and\;\#V\in \left[2,4\right] \end{array}\right) \end{array},$$ (59)

$$\begin{array}{cc} T>20\;\min & if\;\left(\begin{array}{c} {\overset{═}{I}}_{\prime \prime ReDirection\prime \prime }>J+4\\ and\;\#V>4 \end{array}\right) \end{array}.$$ (60)

**TABLE 3:** Run-time characteristics of the “ReDirection”-mediated computation of probable dissociation constants for simulated biochemical networks ( n=50 ).

S. no.	J	${\overset{═}{I}}_{\prime \prime ReDirection\prime \prime }$	#V	$T\left(\min\right)$	Label
1	4	6	2	0.0003	TP
2	4	7	3	0.0003	TP
3	4	8	4	>20	FN
4	4	9	5	>20	TN
5	5	7	2	0.0002	TP
6	5	8	3	0.001	TP
7	5	9	4	>20	FN
8	5	10	5	>20	TN
9	6	8	2	0.0002	TP
10	6	9	3	0.001	TP
11	6	10	4	>20	FN
12	6	11	5	>20	TN
13	7	9	2	0.0002	TP
14	7	10	3	13	TP
15	7	11	4	12.36	TP
16	7	12	5	>20	TN
17	8	10	2	0.0002	TP
18	8	11	3	0.0012	TP
19	8	12	4	>20	FN
20	8	13	5	>20	TN
21	9	11	2	0.0001	TP
22	9	12	3	0.0013	TP
23	9	13	4	15	TP
24	9	14	5	>20	TN
25	10	13	3	0.0011	TP
26	10	14	4	>20	FN
27	10	15	5	>20	TN
28	11	13	2	0.0012	TP
29	11	14	3	0.0012	TP
30	11	15	4	13.6	TP
31	11	16	5	>20	TN
32	13	15	2	0.01	TP
33	13	16	3	0.0013	TP
34	13	17	4	1	TP
35	13	18	5	>20	TN
36	13	19	6	>20	TN
37	14	16	2	0.0012	TP
38	14	17	3	0.07	TP
39	14	18	4	>20	FN
40	14	19	5	>20	TN
41	14	20	6	>20	TN
42	15	17	2	0.0002	TP
43	15	18	3	14	TP
44	15	19	4	>20	FN
45	16	18	3	14	TP
46	16	19	3	>20	FN
47	16	20	4	>20	FN
48	16	21	5	>20	TN
49	17	19	3	>20	FN
50	17	20	4	>20	FN

Abbreviations: J , reactants or products for the user-defined biochemical network; ${\overset{═}{I}}_{\prime \prime ReDirection\prime \prime }$ , reactions considered for computing the probable dissociation constant; #V , cardinality of null space; T, time taken to unambiguously compute the probable dissociation constant for every reaction a user-defined biochemical network; TP, true positive; FP, false positive; FN, false negative; TN, true negative.

On the basis of the time taken by “ReDirection” to complete the annotations for each simulated biochemical network, we can categorize each outcome in terms of the categorical variables $\left(TP,FP,FN,TN\right)$ (Table 3). The complete dataset is summarized as follows:

TP=26, (61)

FP=0, (62)

FN=11, (63)

TN=13. (64)

This yields the following indices to assess our premise:

$$\begin{array}{cc} Precision & =100\%, \end{array}$$ (65)

$$\begin{array}{cc} Recall\;\left(sensitivity\right) & \approx 70\%, \end{array}$$ (66)

$$\begin{array}{cc} Specificity & =100\%, \end{array}$$ (67)

$$\begin{array}{cc} Accuracy & \approx 78\%. \end{array}$$ (68)

Clearly, we can achieve significant proportioning of these data on the basis of our estimate of an upper bound for the reactions of these simulated biochemical networks (accuracy, precision, specificity, and recall). We suggest the following bounds for the number of reactions which a user may specify for a modeled biochemical network:

$${\overset{═}{I}}_{\prime \prime ReDirection\prime \prime }\in \left[J+2,J+4\right].$$ (69)

### 3.3 “ReDirection”-based characterization of physiologically relevant biochemical networks

We now utilize “ReDirection” with these constraints to compute probable dissociation constants and, thence, investigate the biochemical networks of human galactose metabolism and cholesterol biosynthesis.

The presented biochemical network for galactose metabolism comprises a significantly larger fraction $\left(\approx 63\%\right)$ of equivalent reactions, as compared to the forward $\left(\approx 27\%\right)$ and reverse $\left(\approx 10\%\right)$ reactions (Figure 3). We also observe the directional preference of several reactions $\left(\eta_{1}=\eta_{2}\approx 12.7,\eta_{3}\approx 26,\eta_{10}\approx 0.04\right)$ toward the synthesis of UDP-glucose (Figure 3). This, when coupled with the equivalent and sequential conversions to UDP-galactose, lactose, and galactose, $\left(\eta_{4-6}\approx 1.000\right)$ ensures that there is minimal change to the pool of galactose-containing complex carbohydrates and lipids (glycosphingolipids, gangliosides, cerebrosides, and mucopolysaccharides) (Thom et al., 2013; Poulos, 2014). Additionally, since the magnitude of the probable dissociation constant for $\eta_{3}$ is twice that of $\eta_{1}$ and $\eta_{2}$ $\left(\frac{\eta_{3}}{\eta_{1}}=\frac{\eta_{3}}{\eta_{1}}>2.0\right)$ , the utilization of alpha-galactose 1-phosphate is faster than its synthesis. Here, in addition, by the law of mass action, there is a net flux of the network toward the biosynthesis of UDP-glucose (Figure 3). In this scenario, the atypical reactions $\left(r_{12},r_{13}\right)$ function to perturb galactose metabolism with flux toward the synthesis of galactose 1-phosphate $\left(\eta_{12}\approx 13.6\right)$ or galactose $\left(\eta_{13}\approx 12.7\right)$ from UDP-galactose and either complements or compensates, where applicable, reactions $r_{1}$ and $r_{2}$ (Figure 3). These studies suggest a predilection of the biochemical network toward synthesizing galactose, which, along with the activity of UDP-galactose 4-epimerase, constitute a plausible explanation for the milder clinical profile of the inborn errors of galactose metabolism (Raff et al., 1978; Jessen et al., 1985) (Figure 3).

The high number of equivalent reactions $\left(\approx 44\%\right)$ studied for cholesterol biosynthesis in the biochemical network suggests a regulatory role, which may account for the conservation of this pathway across taxa (eukaryotes, bacteria, and archaea) (Figure 4) (Edmond and Popjak, 1974; Nakanishi et al., 1988). Catabolism of the cyclopentanoperhydrophenanthrene (CPPP) ring of cholesterol is elaborate and occurs via the incorporation of a single molecule of oxygen by the heme- and iron-dependent cyclooxygenase P450 monooxygenase system of enzymes. The shunt pathway is a simple and yet an effective way of redirecting mevalonate prior to ring closure (Edmond and Popjak, 1974; Nakanishi et al., 1988; Pappu et al., 2002; Roullet et al., 2012). Smith–Lemli–Opitz syndrome is an inborn error of metabolism that arises due to mutations of the terminal enzyme in cholesterol biosynthesis (delta-7-reductase; EC 1.3.1.21 ) (Pappu et al., 2002; Roullet et al., 2012). This is postulated to result in an increased flux of mevalonate through the shunt pathway along with a concomitant increase in the excretion of urinary mevalonate (Edmond and Popjak, 1974; Nakanishi et al., 1988; Pappu et al., 2002; Roullet et al., 2012). This study supports this notion, at least in theory, with all the probable dissociation constants favoring a prominent role for the shunt pathway $\left(r_{11}\to r_{8}\to r_{7}\to r_{17}\right)$ $\left(\eta_{11}\approx 0.158,\eta_{8}\approx 0.028,\eta_{7}\approx 45,\eta_{17}\approx 9.572\right)$ (Figure 4). The isomeric conversion of isopentenyl pyrophosphate to dimethylallyl pyrophosphate from mevalonate has the greatest numerical value of all the predicted probable dissociation constants $\left(\eta_{7}\approx 45\right)$ for the biochemical network. This, in addition, supports the rapid removal of mevalonate either by conversion (main, shunt) and/or excretion in urine (Edmond and Popjak, 1974; Nakanishi et al., 1988; Pappu et al., 2002; Roullet et al., 2012).

The distribution of equivalent $\left(\approx 53\%;{\overset{═}{I}}_{Heme}=11\right)$ , forward $\left(\approx 37\%;{\overset{═}{I}}_{Heme}=8\right)$ , and reverse ( $\approx 10\%;{\overset{═}{I}}_{Heme}=2$ ) reactions supports a similar inference for heme biosynthesis (Figure 5). The rate-limiting step for heme biosynthesis is the reaction catalyzed by ALAS1 ( δ -aminolevulinate synthetase I; EC 2.3.1.37 ). Our data suggest that the reactions from coproporphyrinogens (I) and (III) to uroporphyrinogens (I) and (III) $\left(\eta_{7}\approx \eta_{10}\approx 0.03\right)$ may also contribute significantly to this self-regulation. These reactions are catalyzed by uroporphyrinogen decarboxylase ( EC 4.1.1.37 ) and uroporphyrins (I) and (III), along with the coproporphyrins (I) and (III) that are subsequently generated by sunlight or the spontaneous removal of protons and can function as organic free radicals (Stein et al., 2017). Additionally, and in the absence of enzyme-catalyzed reactions, this catalytic sequestration of the substrates (uroporphyrinogens (I) and (III) and coproporphyrinogens (I) and (III)) ensures, by the law of mass action, that the flux is toward the biosynthesis of heme and its incorporation into heme proteins (Figure 5). Furthermore, since the free radical cycle involving these is self-propagating, the accumulated products, once generated, considerably damage the neighboring skin and other tissues. This reaction clinically partitions disorders of heme biosynthesis into those with photosensitivity and those with predominantly neuropsychiatric manifestations. Porphyria cutanea tarda (PCT) is an inborn error of metabolism due to a defect in the enzyme uroporphyrinogen decarboxylase and results in debilitating blisters on the skin due to exposure to sunlight (Stein et al., 2017). Our data $\left(\eta_{7}\approx \eta_{10}\approx 0.03\right)$ offer a plausible explanation for the genesis and pathophysiology of PCT (Figure 5).

### 3.4 The probable dissociation constants for a biochemical network are suitable indices of biochemical function

The probable dissociation constants for a biochemical network provides the user with theoretically sound and biochemically relevant indices by which reactions of a biochemical network can be compared along with the corresponding change in the reactants/products (Reinker et al., 2006; Lecca et al., 2009; Haraldsdottir et al., 2012; Shindo et al., 2018; Wittenstein et al., 2022; Kundu, 2023a). A potentially novel application for these data is to incorporate these into simulation studies with the stochastic simulation algorithms (Gillespie, 2007; Kundu, 2016; Kundu, 2021). However, these studies mandate, by definition, the use of every possible reaction during a simulation run. This precludes the direct usage of data that are generated by “ReDirection” since only half the reactions are considered in computing the probable dissociation constants for the modeled biochemical network. The complete set of reactions for a user-defined biochemical network $\left({\overset{═}{I}}_{user}\right)$ is simply

$${\overset{═}{I}}_{user}=2.\left({\overset{═}{I}}_{\prime \prime ReDirection\prime \prime }\right).$$ (70)

We annotate this set of additional half reactions in terms of the probable dissociation constant for the “ReDirection” annotated reaction as (Kundu, 2016; Kundu, 2021; Kundu, 2023a)

$$if\;\eta_{i}\in \left\{\begin{array}{c} R\cap \left(1,\infty \right)\\ R\cap \left(0,1\right)\\ R\cap \left\{1\right\}, \end{array}\right.$$

$$then\;\eta_{-i}=1.0,$$ (Def. 30)

$$for\;the\;pair\;of\;reversible\;reactions\;\left\{r_{i},r_{-i}\right\}.$$

This approach has yielded interesting insights into the export of high-affinity peptides to the plasma membrane by the major histocompatibility complex-I (MHC1) (Kundu, 2021). In that study, the authors examined a low-affinity peptide-driven biochemical network that could also be potentially regulatory and, therefore, important in priming circulating CD8^+^ T-cell lymphocytes into mounting a suitable immune response in the presence of acute and chronic insults (Kundu, 2021). Similarly, a role for reactive oxygen species in facilitating cellular proliferation and transmigration whilst precluding a cell to senescence and apoptosis concomitantly was addressed by creating a biochemical network for an advancing phagocyte toward a noxious stimulus (Kundu, 2016). The transduced signal was modeled to act through lipid raft-interacting actin fibers that could stabilize the actin cytoskeleton of the phagocyte and promote the development of a single dominant lamellipodium in the direction of the noxious stimulus (Kundu, 2016).

## 4 Conclusion

“ReDirection” is an R-package that computes the probable disassociation constant for every reaction of a biochemical network directly from a null space-generated subspace of a stoichiometry number matrix. Whilst mathematical rigor is ensured at all steps, biological relevance is maintained by utilizing parameters and metrics in accordance with established kinetic paradigms. “ReDirection” computes the probable dissociation constant from first principles and can be used to compare biochemical networks under varying intracellular environments (baseline, perturbed), between cells, and across taxa. Although computationally intense and possibly intractable for larger networks, the predictions are reasonably rapid for fewer reactions and are completed quickly in a desktop environment. Future investigations should strive to improve upon computational time, investigate perturbations, and validate some of the findings by simulation studies. “ReDirection” is not discovery-based and is better suited to addressing known and often empirically intractable biochemical problems *in silico* with simulations or generating testable hypotheses in a laboratory setting.

## Data Availability

The original contributions presented in the study are included in the article/Supplementary Material; further inquiries can be directed to the corresponding author.
